# Advancing Plant Breeding with Next-Generation Technologies: Insights from Recent Research

**DOI:** 10.3390/plants13202877

**Published:** 2024-10-15

**Authors:** Seong-Hoon Kim, Inchan Choi, Jung-Bong Kim

**Affiliations:** 1 National Agrobiodiversity Center (Genebank), National Institute of Agricultural Sciences, Rural Development Administration, Jeonju 54875, Republic of Korea; 2 Division of Agricultural Engineering, National Institute of Agricultural Sciences, RDA, Jeonju 54875, Republic of Korea; inchchoi@korea.kr; 3 Institut für Pharmazeutische Biologie, Nussallee 6, 53115 Bonn, Germany; jkim@uni-bonn.de

Genetic resources are the cornerstone of our food supply and play a pivotal role in developing new crop varieties that ensure sustainable agricultural production amid the challenges of climate change [1]. Moreover, genetic resources are fundamental for enhancing the nutritional quality of crops, thereby ensuring that an expanding global population has access to healthy, balanced diets essential for overall well-being [2]. However, despite evolving over several decades, the global system for conserving and utilizing genetic resources still exhibits significant weaknesses and lacks a clear, strategic blueprint. To address these challenges, a Special Issue of *Plants* titled “Next-Generation Research on Plant Genetic Resources: Digital Phenotyping, Genomics, Phenomics, and Phytochemicals” was introduced. This issue comprises six research and review papers that focused on genetic diversity, genome-wide association studies, resistance breeding characteristics, digital phenotyping, gene banks, and genetic resources for agricultural sustainability. Special attention is given to genetic resources and access and benefit-sharing issues, which impede access to the diverse genetic resources essential for plant breeders to fulfill their mission.

The conservation of species and genetic resources seeks to conserve existing genetic diversity while enabling the development and expansion of large genetic resources that meet the needs of current and future uses for both humans and other living species on earth [3]. According to estimates, 15% of the Earth’s land surface is protected for conservation, though this amount varies widely among ecosystems and countries. The cumulative effects of climate change on biodiversity and genetic resources have made conservation more vital than ever [4]. Plant phenotype and genotype can be influenced by environmental conditions, including soil texture, light intensity, and climatic characteristics [5]. High-throughput phenotyping platforms with multiscale resolution facilitate the integration of phenotypic data with other omics data, enabling a comprehensive multi-omics analysis [6].

This Special Issue highlights the genetic diversity and breeding strategies of *Vigna angularis* (Adzuki Beans) [7], focusing on environmental adaptation and early breeding to improve yield strategies using genotyping by sequencing (GBS). The genes related to biotic/abiotic stress resistance and defense within most quantitative trait loci (QTL) for yield-related traits were identified using genome-wide association studies (GWAS). Genomic sliding window analysis of Tajima’s D and a genetic differentiation coefficient (Fst) revealed distinct domestication selection signatures and genotype variations within these QTLs among subpopulations. These findings suggest that biotic/abiotic stressors may lead to selection purging, resulting in the loss of high-yielding genotypes. The study further highlights the significant impact of balancing selection on yield-related traits. Significant genes related to stress resistance impact yield-related traits through balancing selection [7].

Enhanced germplasm data quality through digital phenotyping and improved genetic resource accessibility were studied. The role of RDA-Genebank [8] in germplasm collection, evaluation, conservation, and distribution of diverse plant genetic resources was discussed, and the use of digital phenotyping for plant genetic resources was emphasized. The National Agrobiodiversity Center has been committed to enriching its genetic resource collection, responding to the Nagoya Protocol by supplementing passport data for resources lacking origin information. High-throughput phenotyping techniques have been initiated to enhance germplasm data, starting with seed and root phenotype information stored under agronomic traits. These efforts aim to improve the accessibility and quality of genetic resources for researchers globally.

This Special Issue also explored the digital phenotyping of *Glycine max* (soybean) seeds [9] using RGB imaging and a Python algorithm, with a key focus on seed traits, including length, width, projected area, and aspect ratio. The OpenCV library was used for contour detection to measure these traits, showing a strong correlation between algorithm-derived measurements and those obtained manually or through software applications (SmartGrain and WinDIAS). This contributed to improving seed trait analysis and breeding strategies for high-yield soybean varieties. Focusing on soybean, a genome-wide association study in *Glycine soja* [10] was conducted on protein, oil, and amino acid content. Single-nucleotide polymorphisms (SNPs) were analyzed to enhance the selective breeding programs for soybeans and improve their nutritional value and yield.

The eco-friendly pesticide relationship between the genetic and breeding characteristics of white-backed plant hopper (WBPH) behavior in rice was investigated using rice extracts [11]. The rice extracts effectively deterred WBPH without affecting plant height or resistance gene expression levels. This environmentally friendly pesticide is a promising alternative to chemical control of WBPH.

The conservation of *Allium cepa* (onion) germplasm was reviewed [12], and the critical role of onion germplasm collections in preserving genetic diversity and developing climate-resilient varieties was emphasized. The authors highlighted the importance of international and inter-institutional collaborations in sharing and utilizing onion genetic resources to address future agricultural challenges.

The Special Issue of *Plants* focuses on “next-generation research on plant genetic resources: digital phenotyping, genomics, phenomics, and phytochemicals” and highlights the potential of advanced technologies in plant breeding. It is essential to have novel genomic tools and high-throughput phenotyping techniques, as well as advanced information technologies for analyzing large datasets, to succeed. In modern plant breeding, these technologies facilitate and enhance the efficient and cost-effective conservation and utilization of plant genetic resources.

The various studies cited in this issue demonstrate the importance of germplasm in improving agricultural sustainability. Researchers have gained valuable insights into genetic diversity by using next-generation sequencing (NGS) techniques, resulting in improved yields and resistance to biotic and abiotic stresses. GWAS (genome-wide association studies) have proven to be effective at identifying novel genetic markers and characteristics, as demonstrated by techniques such as genotyping by sequencing (GBS), RGB imaging for digital phenotyping, and genome-wide association studies (GWAS). These techniques accelerate the breeding process, particularly in perennial crops, and contribute to the development of climate-resilient species.

In addition, the issue emphasizes the importance of addressing genetic resources, access, and benefit-sharing issues, which are vital for plant breeders to achieve their goals. The use of high-throughput phenotyping and advanced genomic techniques results in a complete knowledge of genetic resources, facilitating their conservation and optimal use. This Special Issue provides a comprehensive snapshot of the most up-to-date scientific and technological advancements in plant breeding. Producing superior crop varieties requires continual improvement, collaboration, and investment in genomic and phenomic techniques. These advancements are crucial to meeting the growing food demands of a growing population.
